# Ensuring infectious safety of colonoscopy

**DOI:** 10.1186/1753-6561-5-S6-P309

**Published:** 2011-06-29

**Authors:** T Grenkova, E Selkova, V Aleshkin, A Melkumyan

**Affiliations:** 1 MRIEM, Moscow, Russian Federation

## Introduction / objectives

The growing prevalence of infectious diseases among patients, including HIV infection, increases the risk of transmission of an infection during endoscopic procedures. In 2009 the first 3 cases of HIV transmission through colonoscopy were reported in the USA.

## Objective

Studying of infectious danger of colonoscopes, processed in the manual and automated way.

## Methods

Microbiology (determination of total bacterial contamination, lgCFU/ml).

## Materials

Swab samples taken from instrumental channels of colonoscopes immediately after the procedure (sample 1), after mechanical brushing of channels (sample 2), and after completing high-level disinfection (HLD) or a complete reprocessing cyclein the Automatic Endoscope Reprocessor (AER) ASP Johnson & Johnson (sample 3). Swabs taken from 48 colonoscopes were studied.

## Results

Bacterial contamination of instrumental channels (samples 1, 2, and 3) in 17 colonoscopes after their use, brushing, and processing in AER was 8.7 (8-10), 4.74 (3.32-6.3), and 0 lgCFU/ml, respectively. Bacterial contamination (samples 1 and 3) of 16 colonoscopes processed in AER only was 8.87 (8-9.7) and 3.8 (2.7-4.85) lgCFU/ml, respectively. Bacterial contamination (samples 1, 2, and 3) of 15 colonoscopes cleaned manually was 8.99 (8-10.4), 5.2 (3-6.7), and 2.9 (2.7-4.1) lgCFU/ml, respectively.

## Conclusion

Processing of colonoscopes in the AER, after preliminary brushing of channels, is the most reliable and effective. In 2008, we have proved that the endoscopes after use on HIV - infected patients represent essential infectious danger. HIV was isolated in the MT-4 human T-lymphoblastoid cells in 33 of 35 (94.3%) samples taken from instrumental channels of endoscopes directly after use and in three samples (8.6%) after completing HLD. All cases of ineffective cleaning were associated with violations of national standards.

## Disclosure of interest

None declared.

